# Postpartum Depressive Symptoms Among Women with Elevated Prenatal Anxiety in Kazakhstan: Prospective Observational Cohort Study

**DOI:** 10.3390/jcm15155869

**Published:** 2026-07-27

**Authors:** Gulzhanat Aitmurzinova, Arailym Baurzhan, Akmaral Mussakhanova, Gulnar Shalgumbayeva, Balday Issenova, Fatima Bagiyarova, Sayat Tanatarov, Lazzat Yermentayeva, Saparkul Arinova, Naslizat Rakhmatullina, Aida Kassymova

**Affiliations:** 1 Department of Pathological Physiology, Astana Medical University, Astana 010000, Kazakhstanermentaeva.l@amu.kz (L.Y.); rakhmatullina.n@amu.kz (N.R.); 2 Department of Radiation Diagnostics, Center of Nuclear Medicine and Oncology of Abai Region, Semey 071400, Kazakhstan; 3 Department of General Medical Practice, Semey Medical University, Semey 071400, Kazakhstan; gulnar.shalgumbayeva@smu.edu.kz; 4 Department of Nursing, Asfendiyarov Kazakh National Medical University, Almaty 050000, Kazakhstan; isenova.b@kaznmu.kz (B.I.); bagiyarova.f@kaznmu.kz (F.B.); 5 Higher Medical College of the Public Health Department of Almaty, Almaty 050020, Kazakhstan

**Keywords:** postpartum depressive symptoms, prenatal anxiety, perinatal mental health, prospective cohort, Kazakhstan

## Abstract

**Background:** Postpartum depression (PPD) is a common mental health condition, and women with elevated prenatal anxiety are at increased risk. Evidence from Kazakhstan remains limited. This study aimed to describe postpartum depressive symptoms and associated factors among women with elevated prenatal anxiety who received routine perinatal care and participated in the PRISM prenatal psychoeducational program. **Methods:** This prospective single-arm cohort study with postpartum follow-up was conducted in Astana, Kazakhstan. Of 1232 pregnant women screened, 234 women with elevated prenatal anxiety participated in the PRISM program, and 226 completed the postpartum assessment. Because no control group was included, the study was not designed to evaluate the effectiveness of PRISM or establish causal effects. Postpartum depressive symptoms were assessed using the Postpartum Depression Screening Scale–Short Form (PDSS-SF). Descriptive statistics were calculated, and associations with age, parity, and postpartum assessment period were examined using Spearman’s rank correlation and non-parametric tests. **Results:** The mean PDSS-SF score was 20.65 ± 6.00. Using a cut-off score of ≥17, 158 of 226 participants screened positive for clinically significant depressive symptoms, corresponding to 69.9% (95% confidence interval (CI): 63.6–75.5%). Scores were highest among women assessed between 6 weeks and 6 months postpartum (*p* < 0.001). Age showed a weak negative correlation with depressive symptoms, whereas parity was not significantly associated. The most frequently reported symptoms were sleep disturbance, loneliness, and depressed mood. **Conclusions:** A substantial burden of postpartum depressive symptoms was observed in this high-risk cohort. The findings support early identification of prenatal anxiety, continued postpartum screening, and access to appropriate psychological and psychiatric care. Controlled studies are needed to evaluate PRISM and similar preventive programs.

## 1. Introduction

Postpartum depression (PPD) is a common mental health condition affecting women during the postpartum period and represents a significant public health concern worldwide. It is estimated that approximately 10–20% of women experience postpartum depression globally, with even higher rates reported in low- and middle-income countries [1,2]. PPD is associated with a range of adverse outcomes for both mothers and infants, including impaired maternal functioning, difficulties in mother–infant bonding, and negative effects on child development [3,4]. Despite increased recognition, PPD remains underdiagnosed and undertreated, particularly in settings with limited access to mental health services [5].

Perinatal anxiety is another highly prevalent but frequently overlooked condition occurring during pregnancy and the postpartum period. Epidemiological studies estimate that anxiety symptoms affect approximately 15–25% of women during the perinatal period, with prevalence varying depending on assessment methods and population characteristics [6,7]. Accumulating evidence suggests that perinatal anxiety is an important predictor of postpartum depression and may precede its onset. Women experiencing elevated anxiety during pregnancy are at substantially higher risk of developing depressive symptoms after childbirth. This relationship underscores the importance of early identification of high-risk women and supports the implementation of preventive strategies during pregnancy [8,9].

Preventive strategies targeting maternal mental health during pregnancy have gained increasing attention in recent years. Psychoeducational interventions, particularly those grounded in cognitive–behavioral principles and incorporating stress management and emotion regulation techniques, have demonstrated effectiveness in reducing perinatal anxiety and improving psychological outcomes [10,11]. Systematic reviews and meta-analyses indicate that such interventions can significantly decrease symptoms of anxiety and depression during the perinatal period, although heterogeneity across studies remains considerable [11,12]. However, most of the available evidence originates from high-income countries, and there is a lack of research evaluating the effectiveness and implementation of these interventions in low- and middle-income settings [6,13,14].

In Kazakhstan, evidence on perinatal mental health remains limited. In a cross-sectional study of 251 women attending five primary healthcare centers in Semey, 59.4% screened positive for postpartum depression within one year after childbirth using the Edinburgh Postnatal Depression Scale [15]. However, this estimate was derived from a single-city clinical sample and should not be interpreted as nationally representative. Psychological distress has also been documented among pregnant women receiving prenatal care and among Kazakhstani women with recurrent pregnancy loss, but population-based evidence on prenatal anxiety and its relationship with postpartum depressive symptoms remains scarce [16,17]. Population-based evidence on prenatal anxiety and its relationship with postpartum depressive symptoms therefore remains scarce.

PRISM was developed by the research team for the present study as a structured prenatal psychoeducational program for women with elevated prenatal anxiety. It was delivered alongside routine perinatal care by trained clinical psychologists and consisted of six sessions conducted over three weeks, with each session lasting approximately 60–90 min. Sessions were delivered individually or in groups according to participant preference and organizational feasibility. The program incorporated cognitive–behavioral principles, psychoeducation about emotional changes during pregnancy, recognition and management of anxiety, breathing and relaxation techniques, emotional regulation, mindfulness, strengthening of social support, preparation for childbirth and the postpartum period, and development of a longer-term coping plan. Detailed session-by-session content is provided in Supplementary Table S1.

The study was not designed to evaluate the effectiveness of PRISM. Rather, it aimed to describe postpartum depressive symptoms and explore associated factors in a high-risk cohort of women who received routine perinatal care and participated in the PRISM program in Astana, Kazakhstan.

## 2. Methods

### 2.1. Study Design and Setting

This prospective single-arm cohort study with postpartum follow-up was conducted in Astana, Kazakhstan, between June 2025 and February 2026 at the Astana City Perinatal Center and the National Scientific Center for Motherhood and Childhood. The Astana City Perinatal Center is a public specialized maternity facility providing obstetric and neonatal care, including care for high-risk pregnancies and newborns. The National Scientific Center for Motherhood and Childhood is a tertiary-level referral institution providing specialized multidisciplinary care for pregnant women, mothers, and children. Together, the participating facilities serve women from Astana and patients referred from other regions of Kazakhstan. During the study period, the facilities provided care for approximately 1232 pregnant women or deliveries annually.

Women with elevated prenatal anxiety received routine perinatal care and participated in the PRISM prenatal psychoeducational program. The study used a descriptive observational framework and aimed to characterize postpartum depressive symptoms and associated factors in this high-risk cohort. Because no control group was included, the study was not designed to evaluate PRISM effectiveness or establish causal effects.

### 2.2. Participants and Recruitment

Pregnant women receiving care at the participating perinatal facilities in Astana were recruited consecutively during the study period. Inclusion criteria were age ≥18 years, gestational age between 12 and 36 weeks, a PASS-R score of ≥26, willingness to participate, and absence of a documented history of diagnosed mental illness. Women with severe psychiatric disorders or severe somatic conditions that could interfere with participation were excluded.

**Figure 1:**
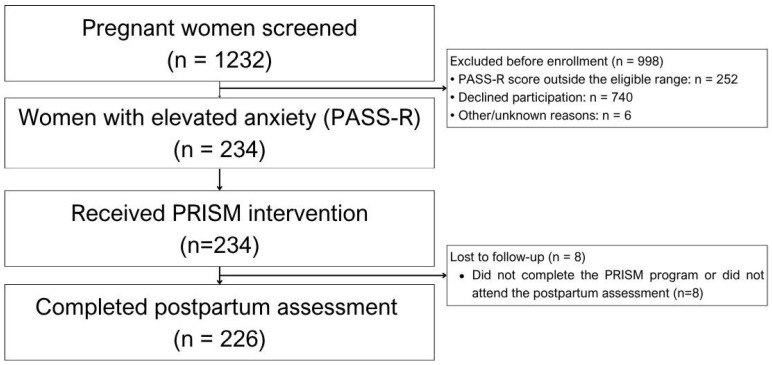
Participant flow diagram.

A total of 1232 pregnant women were approached for screening. Of these, 740 declined participation at different stages of recruitment, including before completion of the screening and eligibility assessment and after being identified as potentially eligible. The numbers declining at each stage were not systematically recorded. A total of 234 eligible women agreed to participate, provided written informed consent, and entered the PRISM prenatal psychoeducational program alongside routine perinatal care. Eight participants did not complete postpartum follow-up because they either discontinued the PRISM program or did not attend postpartum screening. The remaining 226 participants completed the postpartum assessment and were included in the final analysis (Figure 1). The exact distribution of reasons and timing of withdrawal among these eight participants was not systematically recorded.

### 2.3. Sample-Size Justification

No formal a priori sample-size calculation was performed because the study was exploratory and descriptive rather than designed to test intervention effectiveness. The sample size was determined pragmatically by the number of eligible women identified and enrolled consecutively at the participating facilities during the predefined recruitment period. All eligible women who consented to participate were included.

With 226 participants in the final analysis, the observed proportion screening positive for clinically significant postpartum depressive symptoms was 69.9%, with an approximate 95% confidence interval of 63.6–75.5%. This level of precision was considered adequate for the descriptive objective of estimating postpartum symptom burden in this high-risk cohort.

### 2.4. Assessment of Perinatal Anxiety

Prenatal anxiety was assessed before participation in the PRISM program using the Russian-language version of the Perinatal Anxiety Screening Scale (PASS-R). The PASS-R is a 31-item self-report instrument developed to assess a broad range of anxiety symptoms during pregnancy and the first postpartum year. Each item is rated from 0 (“not at all”) to 3 (“almost always”), producing a total score ranging from 0 to 93, with higher scores indicating greater anxiety severity. The instrument assesses four domains: excessive worry and specific fears; perfectionism, control, and trauma; social anxiety; and acute anxiety and adjustment symptoms. A total score of ≥26 was used to identify women at increased risk of clinically significant perinatal anxiety [18,19]. The Russian-language version has demonstrated acceptable validity and reliability [20].

The PASS-R was administered during pregnancy, at 12–36 weeks of gestation, before the first PRISM session and before participants received any psychoeducational intervention.

### 2.5. PRISM Psychoeducational Program

Women identified with elevated prenatal anxiety participated in the structured PRISM prenatal psychoeducational program alongside routine perinatal care. The program was preventive and educational in nature and was based primarily on cognitive–behavioral principles, incorporating psychoeducation, stress-management techniques, emotional regulation, mindfulness, relaxation, and social-support planning.

PRISM consisted of six sessions delivered twice weekly over three weeks, with each session lasting approximately 60–90 min. Sessions were conducted either individually or in groups of approximately 5–10 participants, depending on participant preference, availability, and logistical considerations. A detailed session-by-session description is provided in Supplementary Table S1.

The sessions were delivered by clinical psychologists employed at the participating medical organizations. The intervention combined short educational presentations, guided discussions, breathing and relaxation exercises, mindfulness practice, emotional self-monitoring, role-play, case discussions, and homework assignments. Materials included emotional diaries, guided exercise instructions, support-planning activities, and individualized coping plans.

The program aimed to strengthen coping skills, emotional self-regulation, recognition and management of anxiety symptoms, and preparation for childbirth and the postpartum period. Delivery followed a predefined six-session structure; however, no formal fidelity checklist or independent fidelity assessment was used.

### 2.6. Assessment of Postpartum Depressive Symptoms

Postpartum depressive symptoms were assessed using the Postpartum Depression Screening Scale–Short Form (PDSS-SF), a seven-item version derived from the original 35-item Postpartum Depression Screening Scale developed by Beck and Gable [21,22]. Each item is rated on a five-point Likert scale, producing a total score ranging from 7 to 35, with higher scores indicating greater depressive symptom severity.

A cut-off score of ≥17 was used to identify participants screening positive for clinically significant depressive symptoms [23,24]. This threshold was originally supported by validation of the Portuguese-language PDSS-SF in a postpartum population in Southern Brazil and has subsequently been applied in other postpartum populations. However, the PDSS-SF and the ≥17 threshold have not been independently validated in Kazakhstan or specifically among Russian- or Kazakh-speaking women. Therefore, the results were interpreted as positive screening findings rather than clinical diagnoses of postpartum depression.

Postpartum assessments were conducted at varying time points after delivery because follow-up depended on participants’ availability and attendance at postpartum visits. Logistical challenges, including difficulties contacting some participants and differences in the timing of return visits, prevented assessment at a uniform postpartum time point. Participants were therefore categorized into three postpartum periods: ≤6 weeks, >6 weeks to 6 months, and >6 months. Each participant completed the PDSS-SF once. Prenatal depressive symptoms were not assessed at baseline.

In addition to PASS-R and PDSS-SF scores, the study collected data on maternal age, parity, and postpartum assessment period.

Responses to the item assessing suicidal ideation were more frequently concentrated in the lower response categories than responses to the other PDSS-SF items. However, any endorsement of suicidal thoughts is clinically important and requires prompt individual assessment and appropriate referral. Information on subsequent safety assessments and referrals was not systematically recorded in the study dataset.

### 2.7. Statistical Analysis

Continuous variables were summarized using means and standard deviations or medians and interquartile ranges, as appropriate, while categorical variables were presented as frequencies and percentages. Distributional assumptions were examined using histograms, quantile–quantile (Q–Q) plots, the Shapiro–Wilk test, and skewness statistics. Parametric tests were used for approximately normally distributed variables, whereas non-parametric tests were applied when distributional assumptions were not met.

Each participant completed the PDSS-SF once during the postpartum period. Therefore, the three postpartum-period categories represented independent groups rather than repeated measurements. Differences in PDSS-SF scores across these groups were assessed using the Kruskal–Wallis test. When the overall test was significant, pairwise comparisons were performed using Dunn’s test with Holm adjustment for multiple comparisons. Epsilon-squared (ε^2^) was reported as the effect size for the Kruskal–Wallis comparison.

Parity was operationalized as the total number of children and analyzed as an ordinal/count variable. Associations of PDSS-SF scores with maternal age and parity were evaluated using Spearman’s rank correlation. A 95% confidence interval was calculated for the proportion of participants screening positive on the PDSS-SF. Statistical significance was set at *p* < 0.05.

All statistical analyses were performed using IBM SPSS Statistics for Windows, version 30.0 (IBM Corp., Armonk, NY, USA). Dunn’s post hoc tests with Holm adjustment and epsilon-squared effect sizes were calculated using R version 4.5.1 (R Foundation for Statistical Computing, Vienna, Austria).

### 2.8. Ethical Considerations

The study was conducted in accordance with the principles of the World Medical Association Declaration of Helsinki. Ethical approval was obtained from the Local Bioethical Committee of Astana Medical University (Decision No. 2, Meeting No. 8, dated 9 June 2025). Written informed consent was obtained from all participants prior to enrollment. Confidentiality and anonymity of the collected data were ensured throughout the study.

## 3. Results

### 3.1. Participant Characteristics

A total of 226 women were included in the final analysis. The mean age of participants was 33.5 ± 9.4 years (median: 32, range: 18–47) (Table 1). Regarding the postpartum period, 36 participants (15.9%) were assessed within 6 weeks after delivery, 64 (28.3%) between 6 weeks and 6 months, and 126 (55.8%) more than 6 months postpartum. In terms of parity, 78 participants (34.5%) were primiparous, 79 (35.0%) had two children, and 69 (30.5%) had three or more children.

### 3.2. PDSS-SF Total Score

**Figure 2:**
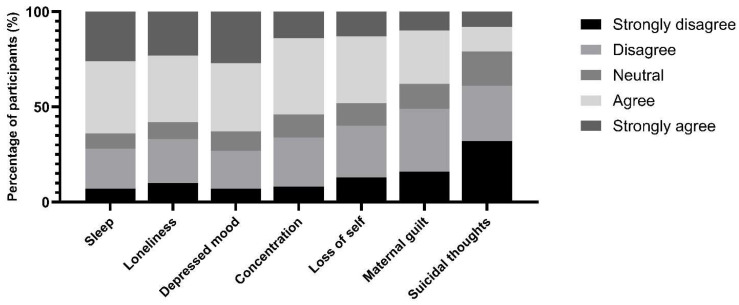
Distribution of responses across Postpartum Depression Screening Scale–Short Form (PDSS-SF) items. Values represent the percentage of participants selecting each response option (*n* = 226).

Among 226 participants, the mean PDSS-SF score was 20.65 ± 6.00, with a median of 21 (range: 7–34). Using a cut-off score of ≥17 to indicate clinically significant depressive symptoms [23,24], 158 participants (69.9%) were classified as having clinically significant depressive symptoms, while 68 participants (30.1%) scored below this threshold. An item-level analysis demonstrated variability in response distributions across the assessed domains (Figure 2 and Supplementary Table S2). Higher proportions of agreement (scores 4–5) were observed for items related to sleep disturbance, loneliness, and depressed mood. Items reflecting cognitive impairment and loss of self showed a more balanced distribution of responses. In contrast, responses to the item assessing suicidal ideation were predominantly concentrated in the lower categories (scores 1–2), indicating lower endorsement relative to other domains.

### 3.3. Additional Analyses

Spearman’s correlation analysis showed no significant association between PDSS-SF scores and number of children (ρ = −0.078, *p* = 0.242). A weak negative correlation was observed between PDSS-SF scores and age (ρ = −0.268, *p* < 0.001).

Each participant completed the PDSS-SF once. Therefore, the postpartum-period categories represented independent groups (Table 2). PDSS-SF scores differed significantly across postpartum periods (Kruskal–Wallis H(2) = 28.66, *p* < 0.001, ε^2^ = 0.120). Dunn’s post hoc test with Holm adjustment showed that participants assessed between 6 weeks and 6 months postpartum had significantly higher scores than those assessed within 6 weeks (adjusted *p* = 0.0045) and those assessed more than 6 months postpartum (adjusted *p* < 0.0001). No significant difference was observed between the ≤6-week and >6-month groups (adjusted *p* = 0.2885).

## 4. Discussion

The present study described postpartum depressive symptoms among women with elevated prenatal anxiety who received routine perinatal care and participated in the PRISM psychoeducational program during pregnancy. A high proportion of participants (69.9%) screened positive for clinically significant depressive symptoms, indicating a substantial psychological burden in this high-risk cohort.

The proportion of participants screening positive for clinically significant depressive symptoms was higher than global estimates of postpartum depression, which generally range from 10% to 20%, and higher than rates reported in many general-population samples from low- and middle-income countries [1,2]. Recent meta-analytic evidence from low- and middle-income countries indicates that postnatal depression estimates vary substantially according to population characteristics and assessment methods, with screening instruments generally producing higher estimates than structured diagnostic interviews [6,25]. This difference likely reflects both the use of a screening measure and the selective inclusion of women with elevated prenatal anxiety rather than broader population-level patterns. Recent evidence also indicates that anxiety and depression frequently co-occur during pregnancy and the postpartum period, supporting the interpretation of prenatal anxiety as a marker of broader psychological vulnerability [26].

In Kazakhstan, available evidence on postpartum mental health remains limited. A previous study conducted in Semey reported a lower proportion of postpartum depression in a general clinical sample than that observed in the present study [15]. The higher proportion identified here may be explained by the deliberate recruitment of women with elevated prenatal anxiety, who already represented a psychologically vulnerable group. Differences in screening instruments, cut-off values, postpartum assessment timing, and sample characteristics may also contribute to variation across studies. Therefore, the present findings should not be interpreted as an estimate of postpartum depression prevalence in the general population of Kazakhstan.

The item-level analysis provided further information about the symptom profile in this cohort. Sleep disturbance, loneliness, and depressed mood were among the most frequently endorsed symptoms. Although sleep disruption is common after childbirth and may partly reflect infant-care demands, its co-occurrence with loneliness and depressed mood may indicate broader postpartum psychological distress. Recent review evidence identifies sleep disturbance, limited social support, childcare-related stress, and previous mental health difficulties among the factors consistently associated with perinatal depressive symptoms [27]. Cognitive impairment, loss of self, and feelings of maternal inadequacy were also reported by a proportion of participants. Suicidal ideation was less frequently endorsed than other symptoms; however, any positive response to an item assessing suicidal thoughts requires careful clinical attention and appropriate referral rather than interpretation solely at the group level.

Exploratory analyses showed a weak negative association between age and PDSS-SF scores, suggesting that younger participants may have been more vulnerable to postpartum psychological distress. This finding should be interpreted cautiously because the association was weak and the study did not adjust for potential confounders such as socioeconomic status, relationship quality, social support, pregnancy complications, or previous psychiatric history. No significant association was observed between parity and depressive symptoms, indicating that the number of children alone may not sufficiently explain postpartum mental health outcomes in this sample.

Depressive symptom levels also differed across postpartum assessment periods, with the highest scores observed among women assessed between 6 weeks and 6 months after childbirth. Population-based evidence indicates that depressive symptoms may emerge or remain clinically relevant later in the postpartum year, supporting continued assessment beyond the early postpartum period [28]. This period may involve increasing caregiving demands, reduced practical support, sleep disruption, and adjustment to maternal responsibilities. Nevertheless, the postpartum groups were composed of different participants rather than repeated assessments of the same women. Therefore, the observed differences cannot be interpreted as an individual symptom trajectory over time. In addition, exact postpartum assessment times were unavailable for some participants, and residual temporal confounding cannot be excluded.

Importantly, although all participants participated in the PRISM psychoeducational program, the study was not designed to evaluate its effectiveness. The absence of a comparison group, baseline depression assessment, and pre–post-measurement of depressive symptoms precludes causal inference and prevents determination of whether symptoms improved, worsened, or remained unchanged following participation. The high proportion of positive PDSS-SF screens should therefore not be interpreted as evidence that PRISM was ineffective or harmful. The cohort was intentionally restricted to women with elevated prenatal anxiety, and some participants may already have experienced coexisting depressive symptoms during pregnancy.

PRISM was a brief psychoeducational program intended to strengthen awareness, coping, emotional regulation, and stress-management skills. It was not designed as a substitute for comprehensive psychiatric or psychological treatment for established depressive disorders. Meta-analytic evidence from randomized studies suggests that psychological interventions can reduce perinatal depressive symptoms, although effects vary according to intervention type, intensity, delivery format, population, and study design [29]. The present study cannot determine whether PRISM produced such effects because it lacked a comparison group and baseline and repeated depression assessments. Psychoeducation alone may be insufficient for women with persistent, moderate-to-severe, or clinically complex symptoms. Such women may require individualized psychiatric assessment, structured psychotherapy, close clinical follow-up, and pharmacological treatment when clinically indicated [30]. The findings therefore support a stepped-care model in which psychoeducation is combined with systematic screening, risk assessment, monitoring, and referral pathways to higher-intensity services.

These findings have implications for clinical practice and public health. Early identification of prenatal anxiety may help recognize women at increased risk of postpartum psychological difficulties. However, screening during pregnancy should be accompanied by continued postpartum assessment because symptoms may emerge or become more prominent after childbirth. Integrating brief psychological support with clear referral pathways could be especially important in settings where specialist perinatal mental health services are limited. Women screening positive for clinically significant depressive symptoms should receive further clinical evaluation because screening results alone do not establish a diagnosis.

This study has several limitations. First, the prospective single-arm cohort design and absence of a comparison group preclude evaluation of PRISM effectiveness and any causal interpretation of the observed postpartum outcomes. In addition, prenatal depressive symptoms were not assessed at baseline, preventing adjustment for an important confounder and limiting the ability to determine whether postpartum symptoms were new-onset, persistent, or worsening.

Second, postpartum assessments were conducted at substantially different time points because of logistical constraints and variation in participant follow-up. Exact assessment times in weeks or months were unavailable for all participants, preventing calculation of the median and range. Although assessment timing categories were included in the analysis, temporal variability may have influenced PDSS-SF scores, and the cross-sectional comparison of independent postpartum groups cannot be interpreted as an individual symptom trajectory.

Third, both anxiety and depressive symptoms were assessed using self-reported screening instruments. The PDSS-SF cut-off has not been validated specifically in Kazakhstan or in Russian- and Kazakh-speaking populations, and cultural or linguistic differences may have affected classification. Screening results should therefore not be interpreted as confirmed clinical diagnoses.

Fourth, information on several potentially relevant factors, including prenatal depression, previous mental health history beyond documented diagnoses, social support, socioeconomic circumstances, obstetric complications, and postpartum treatment, was limited or unavailable. The study was therefore unable to adjust for these potential confounders.

Fifth, although participants were recruited consecutively, participation was voluntary and may have introduced self-selection bias. Eight participants did not complete postpartum follow-up, and detailed information on the distribution and timing of attrition was not systematically recorded, limiting assessment of attrition bias. No formal a priori sample-size calculation was performed because enrollment was determined pragmatically during the predefined recruitment period; consequently, the study may have had limited power for exploratory subgroup and association analyses. Finally, recruitment from two facilities in a single urban setting may limit generalizability to rural populations and other regions of Kazakhstan.

Despite these limitations, the study provides context-specific evidence on postpartum depressive symptom burden among women with elevated prenatal anxiety in Kazakhstan. The findings emphasize the importance of early identification during pregnancy, continued postpartum screening, and access to stepped psychological and psychiatric care. The principal contribution of this study is the description of postpartum depressive symptom burden in a high-risk Kazakhstani cohort rather than evidence regarding intervention effectiveness. Future controlled studies should include baseline and repeated assessments of anxiety and depression, locally validated instruments, broader clinical and social variables, and appropriate comparison groups. Randomized or well-designed comparative studies are needed to evaluate PRISM, while longitudinal research is required to clarify individual symptom trajectories across the perinatal period.

## Figures and Tables

**Table 1:** Participant characteristics (*n* = 226). Abbreviations: *n*, number of participants; SD, standard deviation.

Variable	*n* (%) or Mean ± SD
Age (years)	
Mean ± SD	33.5 ± 9.4
Median (range)	32 (18–47)
Postpartum period	
≤6 weeks	36 (15.9%)
6 weeks–6 months	64 (28.3%)
>6 months	126 (55.8%)
Parity (number of children)	
One child	78 (34.5%)
Two children	79 (35.0%)
Three or more children	69 (30.5%)

**Table 2:** Comparison of Postpartum Depression Screening Scale–Short Form (PDSS-SF)scores across postpartum assessment periods. Abbreviations: IQR, interquartile range; *n*, number of participants.

Postpartum Period	*n*	PDSS-SF, Median (IQR)
≤6 weeks	36	24.0 (19.0–25.0)
>6 weeks to 6 months	64	25.0 (19.5–26.5)
>6 months	126	18.0 (14.0–25.0)

Note: Kruskal–Wallis H(2) = 28.66, *p* < 0.001, ε^2^ = 0.120.

## Data Availability

The datasets generated and analyzed during the current study are not publicly available due to participant confidentiality, but they are available from the corresponding author upon reasonable request.

## Supplementary Materials

The following supporting information can be downloaded at: https://www.mdpi.com/article/10.3390/jcm15155869/s1, Table S1. Structure and content of the PRISM psychoeducational intervention. Table S2. Distribution of responses across PDSS-SF domains (n, %).

## References

[1] Mohamud R.Y.H., Mohamed N.A., Mohamed Y.A., Ali K.Y., Ali A.N., Mohamud S.M., Doğan S., Karahan N., Ahmed S.A., Hashi A.I. (2025). Prevalence and associated factors of postpartum depression symptoms among mothers in Mogadishu, Somalia: A Hospital-based Cross-sectional Study. J. Mood Anxiety Disord..

[2] Khamidullina Z., Marat A., Muratbekova S., Mustapayeva N.M., Chingayeva G.N., Shepetov A.M., Ibatova S.S., Terzic M., Aimagambetova G. (2025). Postpartum Depression Epidemiology, Risk Factors, Diagnosis, and Management: An Appraisal of the Current Knowledge and Future Perspectives. J. Clin. Med..

[3] Stein A., Pearson R.M., Goodman S.H., Rapa E., Rahman A., McCallum M., Howard L.M., Pariante C.M. (2014). Effects of perinatal mental disorders on the fetus and child. Lancet.

[4] Howard L.M., Khalifeh H. (2020). Perinatal mental health: A review of progress and challenges. World Psychiatry.

[5] Rajendran V.R. (2025). KM Postpartum depression among women: A systematic review of prevalence, risk factors, and maternal outcomes. Int. J. Reprod. Contracept. Obstet. Gynecol..

[6] Roddy Mitchell A., Gordon H., Atkinson J., Lindquist A., Walker S.P., Middleton A., Tong S., Hastie R. (2023). Prevalence of Perinatal Anxiety and Related Disorders in Low- and Middle-Income Countries: A Systematic Review and Meta-Analysis. JAMA Netw. Open.

[7] Nielsen-Scott M., Fellmeth G., Opondo C., Alderdice F. (2022). Prevalence of perinatal anxiety in low- and middle-income countries: A systematic review and meta-analysis. J. Affect. Disord..

[8] Osborne L.M., Voegtline K., Standeven L.R., Sundel B., Pangtey M., Hantsoo L., Payne J.L. (2021). High worry in pregnancy predicts postpartum depression. J. Affect. Disord..

[9] Cheng C.-Y., Chou Y.-H., Chang C.-H., Liou S.-R. (2021). Trends of Perinatal Stress, Anxiety, and Depression and Their Prediction on Postpartum Depression. Int. J. Environ. Res. Public Health.

[10] Clinkscales N., Golds L., Berlouis K., MacBeth A. (2023). The effectiveness of psychological interventions for anxiety in the perinatal period: A systematic review and meta-analysis. Psychol. Psychother..

[11] Li X., Laplante D.P., Paquin V., Lafortune S., Elgbeili G., King S. (2022). Effectiveness of cognitive behavioral therapy for perinatal maternal depression, anxiety and stress: A systematic review and meta-analysis of randomized controlled trials. Clin. Psychol. Rev..

[12] Dooley R.L., McAloon J. (2025). The efficacy of antenatal Cognitive Behavioural Therapy for antenatal and postnatal depression: A PRISMA based systematic review and meta-analysis. J. Affect. Disord..

[13] Ng’oma M., Waqas A., Seward N., Honikman S., Mbekwani O., Stewart R.C. (2026). Identification, Prevention, and Treatment of Perinatal Depression in Low- and Middle-Income Countries: A Narrative Review. Biol. Psychiatry.

[14] Elias A., Seward N., Lund C. (2024). Predictors, moderators and mediators of psychological therapies for perinatal depression in low- and middle-income countries: A systematic review. Camb. Prism. Glob. Ment. Health.

[15] Abenova M., Myssayev A., Kanya L., Turliuc M.N., Jamedinova U. (2022). Prevalence of postpartum depression and its associated factors within a year after birth in Semey, Kazakhstan: A cross sectional study. Clin. Epidemiol. Glob. Health.

[16] Kossybakova G., Alibekova R., Crape B., Kuntuganova A. (2022). The impact of depression on satisfaction with prenatal care in Kazakhstan during COVID-19 pandemic. Eur. J. Public Health.

[17] Issakhanova A., Issanov A., Ukybassova T., Kaldygulova L., Marat A., Imankulova B., Kamzayeva N., Almawi W.Y., Aimagambetova G. (2023). Depression, Anxiety, and Stress in Kazakhstani Women with Recurrent Pregnancy Loss: A Case–Control Study. J. Clin. Med..

[18] Somerville S., Dedman K., Hagan R., Oxnam E., Wettinger M., Byrne S., Coo S., Doherty D., Page A.C. (2014). The Perinatal Anxiety Screening Scale: Development and preliminary validation. Arch. Womens Ment. Health.

[19] Somerville S., Byrne S.L., Dedman K., Hagan R., Coo S., Oxnam E., Doherty D., Cunningham N., Page A.C. (2015). Detecting the severity of perinatal anxiety with the Perinatal Anxiety Screening Scale (PASS). J. Affect. Disord..

[20] Korgozha M., Evmenenko A. (2023). Validation and reliability of the Russian version of the Perinatal Anxiety Screening Scale. Minerva Psychiatry.

[21] Beck C.T., Gable R.K. (2000). Postpartum Depression Screening Scale: Development and Psychometric Testing. Nurs. Res..

[22] Beck C.T., Gable R.K. (2001). Further Validation of the Postpartum Depression. Nurs. Res..

[23] Miranda A.R., Scotta A.V., Cortez M.V., Soria E.A. (2025). Two-years mothering into the pandemic: Impact of the three COVID-19 waves in the Argentinian postpartum women’s mental health. PLoS ONE.

[24] Zubaran C., Schumacher M.V., Foresti K., Thorell M.R., Amoretti A., Müller L. (2010). The Portuguese version of the Postpartum Depression Screening Scale-Short Form. J. Obstet. Gynaecol..

[25] Gelaye B., Rondon M.B., Araya R., Williams M.A. (2016). Epidemiology of maternal depression, risk factors, and child outcomes in low-income and middle-income countries. Lancet Psychiatry.

[26] Al-abri K., Edge D., Armitage C.J. (2023). Prevalence and correlates of perinatal depression. Soc. Psychiatry Psychiatr. Epidemiol..

[27] Ou L., Shen Q., Xiao M., Wang W., He T., Wang B. (2025). Prevalence of co-morbid anxiety and depression in pregnancy and postpartum: A systematic review and meta-analysis. Psychol. Med..

[28] Robbins C.L., Ko J.Y., D’Angelo D.V., Salvesen Von Essen B., Bish C.L., Kroelinger C.D., Tevendale H.D., Warner L., Barfield W. (2023). Timing of Postpartum Depressive Symptoms. Prev. Chronic Dis..

[29] Cuijpers P., Franco P., Ciharova M., Miguel C., Segre L., Quero S., Karyotaki E. (2023). Psychological treatment of perinatal depression: A meta-analysis. Psychol. Med..

[30] ACOG, Miller E.S., Metz T., Simas T.A.M., Hoffman M.C., Byatt N., Roussos-Ross K. (2023). Treatment and Management of Mental Health Conditions During Pregnancy and Postpartum: ACOG Clinical Practice Guideline No. 5. Obstet. Gynecol..

