# Supplementary material for: Postpartum Depressive Symptoms Among Women with Elevated Prenatal Anxiety in Kazakhstan: Prospective Observational Cohort Study
Source: J Clin Med. 2026 Jul 27;15(15):5869. doi: 10.3390/jcm15155869 (PMC13467482; doi:10.3390/jcm15155869)
Supplement: Supplementary file 1 [file jcm-15-05869-s001.zip › jcm-4355062-supplementary.pdf]

Supplementary Materials

Supplementary Table S1. Structure and content of the PRISM psychoeducational intervention

Session	Topic	Objectives	Key Components	Homework
1	Introduction to emotional health during pregnancy	To introduce the program and increase awareness of perinatal emotional changes	Psychoeducation on anxiety and depression; group discussion; identification of symptoms	Maintain an emotional diary for one week
2	Recognizing and managing anxiety	To help participants identify anxiety triggers and develop basic coping strategies	Breathing exercises; relaxation techniques; introduction to cognitive restructuring	Practice breathing techniques twice daily
3	Emotional regulation and self-care	To develop emotional awareness and self-regulation skills	Mindfulness exercises; guided meditation; self-reflection techniques	Daily mindfulness practice (10–15 minutes)
4	Building social support	To enhance interpersonal communication and support systems	Role-playing; discussion of support networks; partner involvement strategies	Develop a personal support plan
5	Preparation for childbirth and postpartum period	To reduce fear and increase confidence regarding childbirth and early postpartum period	Psychoeducation; visualization techniques; coping strategies for postpartum challenges	Continue emotional diary and relaxation exercises
6	Program consolidation and evaluation	To reinforce learned skills and evaluate progress	Group discussion; reflection; post-intervention assessment; feedback	Develop a long-term self-care and coping plan

Supplementary Table S2. Distribution of responses across PDSS-SF domains (n, %)

Domain	1 (Strongly disagree)	2 (Disagree)	3 (Neutral)	4 (Agree)	5 (Strongly agree)
Sleep disturbance (Q1)	15 (6.6%)	48 (21.2%)	18 (8.0%)	85 (37.6%)	60 (26.5%)
Emotional distress / Loneliness (Q2)	22 (9.7%)	52 (23.0%)	20 (8.8%)	80 (35.4%)	52 (23.0%)
Depressed mood (Q3)	16 (7.1%)	44 (19.5%)	22 (9.7%)	82 (36.3%)	62 (27.4%)
Cognitive impairment (Q4)	18 (8.0%)	58 (25.7%)	26 (11.5%)	92 (40.7%)	32 (14.2%)
Loss of self / Depersonalization (Q5)	30 (13.3%)	62 (27.4%)	28 (12.4%)	78 (34.5%)	28 (12.4%)
Maternal inadequacy / Guilt (Q6)	36 (15.9%)	74 (32.7%)	30 (13.3%)	64 (28.3%)	22 (9.7%)
Suicidal ideation (Q7)	72 (31.9%)	66 (29.2%)	40 (17.7%)	30 (13.3%)	18 (8.0%)
